# The Association of Established Primary Care with Postoperative Outcomes Among Medicare Patients with Digestive Tract Cancer

**DOI:** 10.1245/s10434-024-16042-w

**Published:** 2024-08-19

**Authors:** Erryk S. Katayama, Razeen Thammachack, Selamawit Woldesenbet, Mujtaba Khalil, Muhammad Musaab Munir, Diamantis Tsilimigras, Timothy M. Pawlik

**Affiliations:** 1 Department of Surgery, The Ohio State University Wexner Medical Center and James Comprehensive Cancer Center, Columbus, OH USA; 2 The Ohio State University College of Medicine, Columbus, OH USA

**Keywords:** Primary care, Established care, Access, Continuity, Textbook outcome, Cancer, Surgery

## Abstract

**Background:**

Primary care (PC) is essential to overall wellness and management of comorbidities. In turn, patients without adequate access to PC may face healthcare disparities. We sought to characterize the impact of established PC on postoperative outcomes among patients undergoing a surgical procedure for a digestive tract cancer.

**Methods:**

Medicare beneficiaries with a diagnosis of hepatobiliary, pancreas, and colorectal cancer between 2005 and 2019 were identified within the Surveillance, Epidemiology, and End Results program and Medicare-linked database. Individuals who did versus did not have PC encounters within 1-year before surgery were identified. A postoperative textbook outcome (TO) was defined as the absence of complications, no prolonged hospital stay, no readmission within 90 days, and no mortality.

**Results:**

Among 63,177 patients, 50,974 (80.7%) had at least one established PC visit before surgery. Patients with established PC were more likely to achieve TO (odds ratio [OR], 1.14; 95% confidence interval [CI], 1.09–1.19) with lower odds for complications (OR, 0.85; 95% CI, 0.72–0.89), extended hospital stay (OR, 0.86; 95% CI, 0.81–0.94), 90-day readmission (OR, 0.94; 95% CI, 0.90–0.99), and 90-day mortality (OR, 0.87; 95% CI, 0.79–0.96). In addition, patients with established PC had a 4.1% decrease in index costs and a 5.2% decrease in 1-year costs. Notably, patients who had one to five visits with their PC in the year before surgery had improved odds of TO (OR, 1.21; 95% CI, 1.16–1.27), whereas individuals with more than 10 visits had lower odds of a postoperative TO (OR, 0.91; 95% CI, 0.84–0.98).

**Conclusion:**

Most Medicare beneficiaries with digestive tract cancer had established PC within the year before their surgery. Established PC was associated with a higher probability of achieving ideal outcomes and lower costs. In contrast, patients with more than 10 PC appointments, which was likely a surrogate of overall comorbidity burden, experienced no improvement in postoperative outcomes.

**Supplementary Information:**

The online version contains supplementary material available at 10.1245/s10434-024-16042-w.

Primary care (PC) is the cornerstone of the health care system. PC offers quintessential services of initial and longitudinal patient care by playing an integral role in maintaining overall wellness, preventive care measures, and early intervention for various illnesses. Despite the profound importance of PC, an estimated 74 million individuals in the United States live in PC shortage areas due to an inequitable distribution of resources and a shortage of more than 50,000 physicians.^1,2^ Areas with a PC shortage disproportionately affect individuals from vulnerable socioeconomic backgrounds such as low-income, rural, or minority populations.^3^ For example, a recent study noted that Black patients had worse healthcare outcomes due to lack of PC access or access to PC providers with less experience or no board certification.^4,5^

Primary care plays an integral role in the management of both oncologic and surgical diagnoses, including regular screening, referral to specialists, multidisciplinary coordination of care, and long-term follow-up.^6–8^ In the context of general surgical care, it has been suggested that established PC may decrease postoperative complications and morbidity due to a more rigorous patient-provider relationship that allows for an increased understanding of situational factors and appropriate treatment coordination of patient-specific risk factors and comorbidities.^9,10^ In turn, established PC may translate into better long-term outcomes including increased life expectancy and improved quality of life. Furthermore, PC may result in lower complication rates and subsequent resource utilization, with lower overall health care care costs, among patients undergoing a surgical procedure.^10^ Patients with lower access to PC can be disparately affected by adverse outcomes, especially rural patient populations who may have relatively lower education and annual income.^3^

While the implications of PC have been examined in a general surgical population, the findings may not be generalizable to the surgical oncology population, which has a complex set of treatment needs. In particular, the relationship between established PC and outcomes among patients receiving gastrointestinal surgery to treat cancer has yet to be fully characterized. Therefore, the objective of the current study was to determine the proportion of surgical oncology patients without established PC among Medicare beneficiaries. In addition, we sought to characterize the impact of established PC on the management of gastrointestinal cancer patients and the likelihood to achieve an “optimal” textbook outcome (TO) after surgical resection of digestive tract cancers.

## Methods

### Data Source

The merged database maintained by the National Cancer Institute’s Surveillance, Epidemiology, and End Results (SEER) program and the Centers for Medicare and Medicaid Services Standard Analytic Files was used to identify the analytic cohort. This database offers detailed patient-level data on both overall claims (demographics, diagnoses, procedures, and expenditures) and cancer-specific clinical variables (e.g., cancer type and stage). Patients were included in the study if they were enrolled in both Medicare parts A and B for at least 1-year before their surgery date, and excluded if they participated in a health maintenance organization.

The International Classification of Diseases 9th and 10th revision codes and the Healthcare Common Procedure Coding System/Current Procedural Terminology codes were used to identify patients who had undergone a procedure for a hepatobiliary, pancreatic, or colorectal cancer between 2005 and 2019 (Table S1). Individual patient residential location was identified using state and county Federal Information Processing System (FIPS) codes. The study was exempt from review by The Ohio State University institutional review board.

### Exposure

Established PC was defined as at least 1 qualifying encounter with a PC provider within the 365 days before surgery. Based on methodology established by Pham et al.^11^ and Roberts et al.,^9^ primary care encounters were determined by Berenson-Eggers Type of Service codes M1A, M1B, and M6 and included claims from provider specialty codes 01 (general practice), 08 (family practice), 11 (internal medicine), 12 (osteopathic manipulative therapy), 38 (geriatric medicine), 60 (public health or welfare agency), and 84 (preventive medicine). Encounters that occurred outside an outpatient setting were excluded using place of service codes 21 (inpatient), 23 (emergency department), 24 (surgery center), 41 and 42 (ambulance), and 81 (independent laboratory).

### Variables

Patient-specific demographic data including age, sex, race/ethnicity, marital status, rurality, and social vulnerability index (SVI) were collected in addition to clinical information such as primary cancer site and American Joint Committee on Cancer stage of cancer. The SVI, approximated by the aforementioned county-level FIPS codes, is maintained by the Centers for Disease Control and Prevention and the Agency for Toxic Substances and Disease Registry and serves as a validated measure of regional vulnerability to external stressors.^12^ The SVI is based on census-level social variables accounting for household composition, socioeconomic status, infrastructure, and minority composition. A lower composite SVI score reflects lower vulnerability, whereas a higher SVI score represents higher vulnerability and has been correlated with adverse surgical outcomes.^13,14^

Hospital volume was calculated according to specific procedure volume corresponding to each primary cancer type (e.g., colectomy for colon cancer) by dividing the total number of applicable procedures performed in the hospital during the study period by the number of years the hospital performed the procedures. For the purposes of comparison during analysis, the SVI score and the hospital procedure-specific surgical volume were categorized into tertiles (i.e., low, medium, and high) and treated as ordinal instead of continuous variables.

The primary outcome was the achievement of TO, a validated composite measure of favorable results during and after surgical intervention.^15,16^ Textbook outcome was defined as the absence of adverse events during the perioperative period, namely, no postoperative surgical complications, no procedure-specific hospital stays below the 75th percentile, no readmission within 90 days post-discharge, and no 90-day postoperative mortality.^17^ Additionally, discharge disposition (home/self-care, home health agency, skilled nursing facility, or other) was recorded. Health care costs were a secondary outcome, estimated on the basis of payments made by Medicare as well as by those falling under patient responsibility for the index hospitalization. These costs were adjusted using the wage index for regional variation and the Health Care Price Index for inflation.

### Statistical Analysis

Categorical variables are represented in terms of frequencies and percentages, whereas continuous variables are described using median values with interquartile ranges. Assessment of categorical variables involved chi-square or Fisher’s exact tests, whereas continuous variables were compared using the Wilcoxon rank-sum test. Multivariable mixed-effects logistic regression models were used to investigate the relationship between established PC and TO, together with its component elements such as complications, length of hospital stay, readmission, and mortality.

The following covariates were adjusted for in the models: race, sex, region, metropolitan status, SVI, comorbidity status, surgical site, hospital volume, and urgent versus elective. A multivariable sub-analysis was performed to examine the relationship between number of PC appointments in the year before surgery. Patients were categorized into 0, 1 to 5, 6 to 10, and more than 10 appointments. To examine the impact of established PC on expenditures in the index hospitalization and 1-year post-discharge settings, a multivariable generalized linear regression model with a gamma distribution and logit link was used. Analyses were stratified by the type of surgery, and all statistical analyses were performed using SAS version 9.4 (SAS Institute, Cary, NC, USA) with a significance level set at an α value of 0.05.

## Results

### Patient Characteristics

Among 63,177 Medicare beneficiaries who underwent surgical resection of a primary digestive tract cancer (hepatobiliary [*n* = 2954, 4.7%), pancreatic (*n* = 4464, 7.1%], colorectal [*n* = 55,728, 88.2%]), 35,681 (56.5%) were women with a median age of 77 years (interquartile range [IQR] 72–83 years). The majority of the cohort was White (*n* = 51,098, 80.9%), with smaller subsets of patients that identified as Black (*n* = 4375, 6.9%), Hispanic (*n* = 3836, 6.1%), or other race/ethnicity (*n* = 3868, 6.1%). Most patients resided in metropolitan areas (*n* = 52,760, 83.5%) with varying levels of social vulnerability (low [*n* = 20,940, 33.4%], intermediate [*n* = 20,966, 33.4%], high [*n* = 20,836, 33.2%]).  Hospitals varied relative to site-specific volumes for each procedure type (low [n=20,980, 33.2%], intermediate [*n* = 20,918, 33.1%, high [*n* = 21,279, 33.7%]). Final pathology demonstrated various stages of localized disease (stage I [*n* = 17,017, 26.9%], stage II [*n* = 25,680, 40.6%], stage III [*n* = 20,480, 32.4%]).

Whereas most of the cohort had established PC (*n* = 50,974, 80.7%), 12,203 (19.3%) did not have a documented encounter with PC in the year before the index surgical procedure. Among patients without a prior PC encounter, treatment of colorectal cancer was relatively more common (no PC [91.2%] vs PC [87.5%]), whereas pancreatic cancer (no PC [4.5%] vs PC [7.7%]) and hepatobiliary cancer (no PC [5.7%] vs PC [6.3%]) were less common. Furthermore, pancreatic and hepatobiliary cancers were slightly more likely to present at advanced stages: stage I (no PC [24.0%] vs PC [27.6%]) versus stage III (no PC [35.6%] vs PC [32.6%]) (all *p* < 0.001). Notably, patients without a prior PC visit more frequently were male (no PC [47.0%] vs PC [42.7%]), identified as of a racial/ethnic minority (no PC [22.1%] vs PC [18.4%]), resided in a high social vulnerability area (no PC [35.6%] vs PC [32.6%]) or rural area (no PC [26.5%] vs PC [14.1%]) and were treated at hospitals with a lower hospital volume (no PC [40.9%] vs PC [31.4%]) (all *p* < 0.001; Table 1).

**Table 1:** Clinicodemographic characteristics of cancer patients with and without established primary care (PC)

Characteristic	Total	No prior PC	Established PC	
	(*n* = 63,177) *n* (%)	(*n* = 12,203) *n* (%)	(*n* = 50,974) *n* (%)	*P* Value
Median age: years (IQR)	77 (72–83)	77 (71–83)	78 (72–83)	< 0.001
*Sex*				
Female	35,681 (56.5)	6471 (53.0)	29,210 (57.3)	< 0.001
Male	27,496 (43.5)	5732 (47.0)	21,764 (42.7)	
*Race*				
White	51,098 (80.9)	9505 (77.9)	41,593 (81.6)	< 0.001
Black	4375 (6.9)	1080 (8.9)	3295 (6.5)	
Hispanic	3836 (6.1)	968 (7.9)	2868 (5.6)	
Other	3868 (6.1)	650 (5.3)	3218 (6.3)	
*Social vulnerability*				
Low	20,940 (33.4)	3806 (31.4)	17,134 (33.8)	< 0.001
Intermediate	20,966 (33.4)	3996 (33.0)	16,970 (33.5)	
High	20,836 (33.2)	4307 (35.6)	16,529 (32.6)	
Unknown	435	94	341	
*Residential area*				
Metropolitan	52,760 (83.5)	8965 (73.5)	43,795 (85.9)	< 0.001
Rural	10,414 (16.5)	3235 (26.5)	7179 (14.1)	
Hospital volume				
Low	20,980 (33.2)	4989 (40.9)	15,991 (31.4)	<0.001
Intermediate	20,918 (33.1)	3873 (31.7)	17,045 (33.4)	
High	21,279 (33.7)	3341 (27.4)	17,938 (35.2)	
*Cancer site*				
Hepatobiliary	2985 (4.7)	691 (5.7)	2361 (6.3)	< 0.001
Pancreatic	4464 (7.1)	544 (4.5)	3920 (7.7)	
Colorectal	55,728 (88.2)	11,135 (91.2)	44,593 (87.5)	
*Cancer stage*				
I	17,017 (26.9)	2933 (24.0)	14,084 (27.6)	< 0.001
II	25,680 (40.6)	5066 (41.5)	20,614 (40.4)	
III	20,480 (32.4)	4204 (34.5)	17,276 (31.9)	
*Chronic comorbidity*				
Any	6003 (9.5)	1312 (10.8)	4691 (9.2)	< 0.001
Heart disease	3298 (5.2)	704 (5.8)	2594 (5.1)	
COPD	1162 (1.8)	279 (2.3)	883 (1.7)	
Diabetes mellitus	3223 (5.1)	689 (5.6)	2534 (5.0)	
Alzheimer’s	109 (0.2)	34 (0.3)	75 (0.1)	
CKD	1407 (2.2)	296 (2.4)	1111 (2.2)	

IQR, interequartile range; COPD, chronic obstructive pulmonary disease; CKD, chronic kidney disease

### Postoperative Outcome

The incidence of TO in the overall cohort was 54.3%. Patients without established PC were less likely to achieve a textbook postoperative outcome (no PC [49.6%] vs PC [55.4%]), with corresponding increased risk of adverse outcomes relative to the individual TO components including complications (no PC [19.4%] vs PC [16.4%]), prolonged hospital stay (no PC [28.2%] vs PC [21.6%]), 90-day readmission (no PC [28.4%] vs PC [26.4%]), and 90-day mortality (no PC [5.2%] vs PC [4.2%]) (all *p* < 0.001). Furthermore, patients without established PC were less frequently discharged to independent care at home (no PC [49.0%] vs PC [52.9%]), and instead had a higher incidence of skilled nursing facility usage (no PC [25.7%] vs PC [21.9%]) (*p* < 0.001). Patients without PC were more likely to undergo urgent surgery (no PC [43.5%] vs PC [30.3%]) instead of elective surgery (Table S2).

**Fig. 1:**
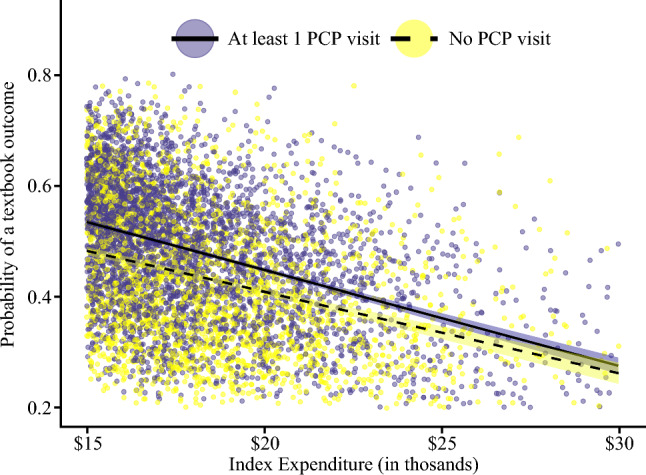
Risk-adjusted index surgery-related hospitalization expenditures among patients with and without established primary care in relation to textbook outcome rates

In addition to higher risk of adverse outcomes, perioperative care of patients without established PC was associated with higher health care expenditures for the index surgical admission (no PC: $12,517 [95% CI, $9437–$16,945] vs PC: $11,991: [95% CI, $8874–$14,826]; *p* < 0.001), as well as the 1-year postoperative period (no PC: $7,082 [95% CI, $1059–$21,084] vs PC: $5809 [95% CI, $798–$18,602]; *p* < 0.001). Specifically, established PC was associated with a 4.1% decrease in index costs (*p* < 0.001) and a 5.2% decrease in 1-year costs (*p* = 0.0003) (Fig. 1). Of note, for any given expenditure amount, patients with established PC had a higher likelihood of TO than individuals without an antecedent PC encounter.

**Fig. 2:**
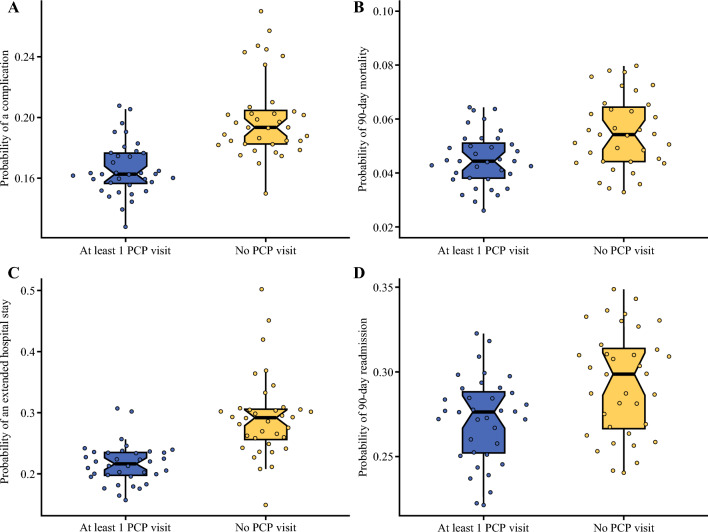
Adjusted probability of achieving each textbook outcome component, stratified by primary care status. **A** Complications. **B** 90-Day mortality. **C** Extended hospital stay. **D** 90-Day readmission

A multivariable analysis further demonstrated the independent association of established PC with better postoperative outcomes. At least one visit with PC in the year before surgery was associated with increased odds for TO (OR, 1.14; 95% CI, 1.09–1.19; *p* < 0.001), with subsequent decreased odds for complications (OR, 0.89; 95% CI, 0.72–0.85; *p* < 0.001) and prolonged hospital stay (OR, 0.81; 95% CI, 0.86–0.94; *p* < 0.001), as well as readmission (OR, 0.94; 95% CI, 0.90–0.99; *p* = 0.0103) or mortality (OR, 0.87; 95% CI, 0.79–0.96; *p* = 0.0037) within 90 days (Table 2; Fig. 2).

**Table 2:** Multivariable analysis of postoperative outcomes among surgical patients with any prior PCP visits versus no prior PCP visits

Outcome	OR	95% CI	*p* Value
Textbook outcome	1.14	1.09–1.19	< 0.001
Complication	0.85	0.72–0.85	< 0.001
Prolonged hospital stay	0.86	0.81–0.94	< 0.001
90-Day readmission	0.94	0.90–0.99	0.0103
90-Day mortality	0.87	0.79–0.96	0.0037

PVP, primary care provider; OR, odds ratio; CI, confidence interval

To further examine the “dose effect” of number of PC appointments relative to outcomes, the multivariable analysis was stratified by number of visits with a PC provider in the year before surgery while still adjusting for competing risk factors. Patients without comorbidities were more likely to have one to five prior visits, whereas individuals with a larger comorbidity burden were more likely to visit their PC more then five times or not at all (*p* < 0.001; Table S3). Compared with patients who had no PC visits, individuals who had seen their PC provider one to five times in the last year had improved outcomes, including higher odds of a TO (OR, 1.21; 95% CI, 1.16–1.27) and lower odds of complications (OR, 0.85; 95% CI, 0.81–0.90), prolonged hospital stay (OR, 0.79; 95% CI, 0.75–0.83), 90-day readmission (OR, 0.88; 95% CI, 0.83–0.92), and 90-day mortality (OR, 0.83; 95% CI, 0.75–0.92) (all *p* < 0.001). In contrast, patients who had more than 10 visits experienced worse outcomes with lower odds to achieve TO (OR, 0.91; 95% CI, 0.84–0.98; *p* = 0.0110) and higher risk of 90-day readmission (OR, 1.23; 95% CI, 1.14–1.33; *p* < 0.001). Patients with more than 10 visits had a comparable risk of complications (OR, 1.02; 95% CI, 0.93–1.12; *p* = 0.7068), prolonged hospital stay (OR, 0.93; 95% CI, 0.85–1.01; *p* = 0.0926), or 90-day mortality (OR, 0.99; 95% CI, 0.84–1.16; *p* = 0.9045) (Table 3).

**Table 3:** Multivariable analysis of postoperative outcomes among surgical patients with established primary care (PC) visits versus no prior PC visits, stratified by number of PC appointments in the year before surgery

Outcome	OR	95% CI	*p* Value
*Textbook outcome*			
None	Ref	–	–
1–5	1.21	1.16–1.27	**< 0.001**
6–10	1.05	1.00–1.11	0.0553
>10	0.91	0.84–0.98	**0.0110**
*Complication*			
None	Ref	–	–
1–5	0.85	0.81–0.90	**< 0.001**
6–10	0.95	0.89–1.02	0.1454
>10	1.02	0.93–1.12	0.7068
*Prolonged hospital stay*			
None	Ref	–	–
1–5	0.79	0.75–0.83	**< 0.001**
6–10	0.84	0.79–0.89	**< 0.001**
>10	0.93	0.85–1.01	0.0926
*90-Day readmission*			
None	Ref	–	–
1–5	0.88	0.83–0.92	**< 0.001**
6–10	1.02	0.97–1.08	0.4563
>10	1.23	1.14–1.33	**< 0.001**
*90-Day mortality*			
None	Ref	–	–
1–5	0.83	0.75–0.92	**0.0004**
6–10	0.89	0.79–1.01	0.0631
>10	0.99	0.84–1.16	0.9045

OR, odds ratio; CI, confidence interval

Significant *p*-values <0.05 are bolded

In a sub-analysis stratified by comorbidity status, there was no dose-response or association between more than 10 PC visits and TO. Additionally, having more than 10 PC visits was associated with increased odds of 90-day readmission regardless of comorbidity status (none: OR, 1.16; 95% CI, 1.06–1.28 vs present: OR, 1.22; 95% CI, 1.06–1.42) and a prolonged hospital stay among the patients without comorbidities (OR, 0.87; 95% CI, 0.78–0.97). Overall, the general trends remained consistent in both the unstratified and stratified analyses (Table S4).

## Discussion

Primary care plays a crucial role in the management of all aspects of patient care, including cancer prevention, detection, and intervention. Access to these essential services may not be possible for all patients because PC shortages of 50,000 physicians disadvantage over 74 million Americans.^1,2^ Previous studies have suggested that established and consistent use of PC services before surgery may improve the odds for favorable postoperative outcomes.^9,10^ The current study was important as we specifically examined the impact that established PC had on individuals undergoing surgical interventions for hepatopancreatobiliary and colorectal malignancies. Notably, in the current study, roughly four in five Medicare beneficiaries who underwent surgery for digestive tract cancer had a least one visit with a generalist outpatient PC provider in the year before the index surgical procedure. Patients who lacked established PC were more likely to be a racial/ethnic minority, live in a rural area with high social vulnerability, and receive treatment at hospitals with a lower procedural volume. Established PC was associated with an increased likelihood to achieve optimal postoperative results and be associated with reduced expenses. Nevertheless, individuals with numerous PC appointments (>10/year) did not experience a benefit from more PC encounters, likely due to the fact that the extreme number of PC visits was a surrogate for higher baseline patient comorbidities.

Social determinants of health may shape access to care, a factor known to play a role in health care disparities. Moreover, social vulnerability has been linked to worse surgical outcomes across a range of medical specialties.^13,15,18,19^ In the current study, patients who lacked established PC were more likely to be a racial/ethnic minority (no PC [22.1%] vs PC [18.4%]), socially vulnerable (no PC [35.6%] vs PC [32.6%]), or live in a rural area (no PC [26.5%] vs PC [14.1%]) (Table 1). These findings are consistent with previous research highlighting disparities in access to health care among vulnerable populations. For example, rural areas face significant access disparities due to cultural, educational, financial, and infrastructure limitations, resulting in poorer health outcomes, a higher comorbidity burden, and challenges recruiting health care professionals and maintaining related services.^20,21^ Similarly, minority patients are more likely to lack access to PC providers than White patients; in addition, PC providers caring for minority patients are less inclined to adopt comprehensive preventive measures or possess board certification.^4,5^ As a result, rural, minority, and socially vulnerable patients receive fewer preventive services (i.e., vaccination or comorbidity screening) despite equivalent insurance enrollment, and thus may be at a higher risk for adverse events.^22–24^ To this point, our own group previously noted that patients with high social vulnerability are more likely to experience fragmentation of surgical care.^25^

Established PC has been associated with improved perioperative outcomes. For example, a previous study by Roberts et al.^9^ demonstrated that patients with PC exposure had lower mortality after emergency general surgery in both the in-hospital and the postoperative settings. Likewise, Hyer et al.^10^ demonstrated that preoperative continuity of care (defined as at least 4 outpatient appointments) in the year before hepatopancreatobiliary surgery was associated with lower risk for readmission and lower surgical-related health care expenditures. In the current study, patients with established PC were 14% more likely to achieve a TO (OR, 1.14) even after adjusting for confounding variables; in addition, patients with established PC had a corresponding lower risk of complications (OR, 0.89), extended hospital stay (OR, 0.81), readmission (OR, 0.94), and mortality (OR, 0.87) (Table 2). The benefits of established PC may occur through various multifactorial mechanisms. In terms of primary prevention, PC providers can influence patients to adopt lifestyle changes that mitigate risky behaviors, thereby decreasing the chance of cancer development.^26^ Among patients with cancer, PC is crucial to earlier detection and timely referral for therapy, as well as management of non-cancer morbidities to limit treatment related comorbidity.^27,28^ In the current study, individuals with established PC had a slightly higher incidence of pancreatic and hepatobiliary cancer diagnoses, as well as a tendency toward a lower disease stage upon presentation and intervention. There are inherent challenges to early detection, and many patients have nonspecific subtle symptoms especially with the initial stages of disease. In turn, lack of PC evaluation on a regular basis may lead to delayed presentation.^29,30^

Moreover, beyond the impact on in-hospital outcomes, established PC also was associated with discharge disposition and health care expenses. Notably, patients lacking PC were less inclined to be discharged to home based on their own independent self-care and were more prone to require advanced care such as skilled nursing (Table S1). The higher resource utilization related to management of adverse postoperative events, as well as the increased need for advanced care resulted in increased health care expenditures among patients who lacked PC in the year prior to surgery. In particular, PC was associated with a 4% to 5% reduction in expenditures during the perioperative period (Fig. 1). Beyond cancer, continuity of care and consistent management of chronic conditions such as congestive heart failure, chronic obstructive pulmonary disease, and diabetes mellitus has been linked to reduced resource utilization, as evidenced by decreased rates of hospitalization, emergency department visits, and complications.^31^ Hyer et al.^10^ and Hussain et al.^32^ conducted two studies illustrating that maintaining continuity in established primary and surgical care led to a 12% and 20% decrease in annual total costs linked to the operative treatment of hepatopancreatic and colorectal cancers, respectively. These trends remain consistent across various surgical subspecialties, such as orthopaedic management of shoulder pathology.^33^ Without effective patient discharge instructions and counseling, the responsibility of long-term management can fall on PC services. For instance, patients experiencing common wound-related symptoms after a laparoscopic cholecystectomy frequently sought care at PC clinics rather than attending surgical outpatient follow-up visits, thereby imposing a considerable burden on these community services.^34^

The current study should be interpreted in the context of several limitations. As with any a retrospective study, data maintained in the registries may contain inherent misclassification and reporting biases.^35^ The use of the Medicare dataset inherently limited the analysis to individuals 65 years old or older enrolled in Medicare, whereas the SEER database includes only data from specific counties and states. For example, trends by hospital volume in SEER-participating hospitals may not be representative of other facilities nationally. As a result, the findings might not be applicable to a nationwide younger demographic with private insurance, thus restricting the generalizability of the results. Moreover, our findings could have been affected by self-selection bias. Patients who have established PC may be more inclined to engage with practices that facilitate earlier detection and adopt beneficial preventive lifestyles, such as exercise, good diet, and avoidance of alcohol or smoking.^36^ Conversely, patients who avoid or cannot use PC may delay seeking necessary cancer care, potentially predisposing them to disease progression and poorer postoperative outcomes. Furthermore, patients without established PC likely had more undiagnosed or undocumented comorbidities that may not have been fully captured by the claims data yet still complicated the postoperative course. Similarly, patients who had a single visit to PC solely for preoperative medical clearance might have been classified under “established care” despite not receiving comprehensive care. However, this differentiation could not be made using retrospective coding data. Although incorporating the SEER data enhances the fundamental oncologic clinical understanding, encompassing disease stage and severity, reliance on claims data might not comprehensively capture patient-specific intricacies in care, such as surgical factors and detailed comorbidity status.

In conclusion, one in five patients with digestive tract cancer lacked established PC within the year before surgical resection. Patients without established PC had later-stage disease diagnosed, often resided in rural areas, and were treated at hospitals with lower surgical volume. Established PC was associated with a higher probability of achieving ideal postoperative outcomes and lower costs. In contrast, patients with a very high number PC appointments (>10/year) experienced no improvement or even worse outcomes, likely due to these patients having more comorbidites that necessitated more frequent PC visits. Initiatives to improve access to care may improve prevention measures, multidisciplinary coordination of oncologic services, as well as postoperative surgical outcomes.

## Supplementary Information

Below is the link to the electronic supplementary material.

Supplementary file1 (DOCX 21 kb)
