# Supplementary material for: The Association of Established Primary Care with Postoperative Outcomes Among Medicare Patients with Digestive Tract Cancer
Source: Ann Surg Oncol. 2024 Aug 19;31(12):8170–8. doi: 10.1245/s10434-024-16042-w (PMC11467066; doi:10.1245/s10434-024-16042-w)
Supplement: Supplementary file 1 — Supplementary file1 (DOCX 21 kb) [file 10434_2024_16042_MOESM1_ESM.docx]

**Supplemental Table 1**. International Classification of Disease 9^th^ and 10^th^ edition codes used to identify procedure sites for study cohort.

| **Procedure Site** | **ICD-9 Codes** | **ICD-10 Codes** |
| --- | --- | --- |
| Liver | '5022' '503' | '0FB00ZZ' '0FB03ZZ' '0FB04ZZ' '0FB10ZZ' '0FB13ZZ' '0FB14ZZ' '0FB20ZZ' '0FB23ZZ' '0FB24ZZ' '0FT10ZZ' '0FT14ZZ' '0FT20ZZ' '0FT24ZZ' |
| Gallbladder | '5124' '5123' '5122' '5121' | '0FB40ZZ' '0FB43ZZ' '0FB44ZZ' '0FT40ZZ' '0FT44ZZ' |
| IHC | '5169' | '0FT50ZZ' '0FT54ZZ' '0FT60ZZ' '0FT64ZZ' '0FB50ZZ' '0FB53ZZ' '0FB54ZZ' '0FB60ZZ' '0FB63ZZ' '0FB64ZZ' |
| EHC | '5161' '5163' | '0FT80ZZ' '0FT84ZZ' '0FT90ZZ' '0FT94ZZ' '0FB80ZZ' '0FB83ZZ' '0FB84ZZ' '0FB90ZZ' '0FB93ZZ' '0FB94ZZ' |
| Pancreas | '5222' '5251' '5252' '5253' '5259' '526' '527' '5209' '5296' '5299' | '0F5G0ZZ' '0F5G3ZZ' '0FBG0ZZ' '0FBG3ZZ' '0FTG0ZZ' '0FTG4ZZ' |
| Ampulla | '5162' | '0FTC0ZZ' '0FTC4ZZ' |
| Colon | "1732" "1733" "1734" "1735" "1736" "1739" "4571" "4572" "4573" "4574" "4575" "4576" "4579" "4581" "4582" "4583" | "0DTH4ZZ" "0DTF4ZZ" "0DTL4ZZ" "0DTG4ZZ" "0DTN4ZZ" "0DBE4ZZ" "0DBF4ZZ" "0DBG4ZZ" "0DBH4ZZ" "0DBK4ZZ" "0DBL4ZZ" "0DBM4ZZ" "0DBN4ZZ" "0DTK4ZZ" "0DTM4ZZ" "0DBE0ZZ" "0DBE3ZZ" "0DBE7ZZ" "0DTH0ZZ" "0DTH7ZZ" "0DTF0ZZ" "0DTF7ZZ" "0DTK0ZZ" "0DTL0ZZ" "0DTL7ZZ" "0DTG0ZZ" "0DTG7ZZ" "0DTN0ZZ" "0DTN7ZZ" "0DBE8ZZ" "0DBF0ZZ" "0DBF3ZZ" "0DBF8ZZ" "0DBG0ZZ" "0DBG3ZZ" "0DBG8ZZ" "0DBH0ZZ" "0DBH3ZZ" "0DBH8ZZ" "0DBK0ZZ" "0DBK3ZZ" "0DBK8ZZ" "0DBL0ZZ" "0DBL3ZZ" "0DBL8ZZ" "0DBM0ZZ" "0DBM3ZZ" "0DBM8ZZ"  "0DBN0ZZ" "0DBN3ZZ" "0DBN8ZZ" "0DTK7ZZ" "0DTK8ZZ" "0DTM7ZZ" "0DTM8ZZ" "0DTE0ZZ" "0DTE7ZZ" |
| Rectum | '4835' '4842' '4843' '4849' '4850' '4851' '4859' '4862' '4863' '4865' '4869' | '0DBP3ZZ' '0DBP7ZZ' '0DBP8ZZ' '0DTP4ZZ' '0DTP0ZZ' '0D1N0Z4' '0DTP7ZZ' '0DTP8ZZ' '0D1N4Z4' '0DBP0ZZ' '0DBP4ZZ' |

**Supplemental Table 2**. Frequency table of patient outcomes.

| **Characteristic** | **Total** | **No Prior PC** | **Established PC** |  |
| --- | --- | --- | --- | --- |
|  | **n=63,177** | **n=12,203** | **n=50,974** | **p-value** |
| Postoperative Outcome |  |  |  |  |
| Textbook Outcome | 34,319 (54.3%) | 6,056 (49.6%) | 28,263 (55.4%) | <0.001 |
| Complications | 10,718 (17.0%) | 2,371 (19.4%) | 8,347 (16.4%) | <0.001 |
| Extended Length of Stay | 14,436 (22.9%) | 3,441 (28.2%) | 10,995 (21.6%) | <0.001 |
| 90-day Readmission | 16,926 (26.8%) | 3,461 (28.4%) | 13,465 (26.4%) | <0.001 |
| 90-day Mortality | 2,765 (4.4%) | 637 (5.2%) | 2,128 (4.2%) | <0.001 |
| Discharge Disposition |  |  |  |  |
| Home | 32,948 (52.2%) | 5,980 (49.0%) | 26,968 (52.9%) | <0.001 |
| Home Health Agency | 13,654 (21.6%) | 2,385 (19.5%) | 11,269 (22.1%) | <0.001 |
| Skilled Nursing Facility | 14,301 (22.6%) | 3,132 (25.7%) | 11,169 (21.9%) | <0.001 |
| Other | 2,274 (3.6%) | 706 (5.8%) | 1,568 (3.1%) | <0.001 |
| Surgical Urgency |  |  |  |  |
| Elective | 42,374 (67.1%) | 6,881 (56.5%) | 35,493 (69.7%) | <0.001 |
| Urgent | 20,741 (32.9%) | 5,307 (43.5%) | 15,434 (30.3%) | <0.001 |

**Supplemental Table 3.** Frequency table of number of primary care appointments in the year prior to surgery stratified by comorbidity status.

| **Number of Visits** | **Total** | **No Comorbidities** | **Comorbidities** |  |
| --- | --- | --- | --- | --- |
|  | **n=63,177** | **n=50,850** | **n=12,327** | **p-value** |
| None | 12,203 (19.3%) | 9,679 (19.0%) | 2,524 (20.5%) | <0.001 |
| 1-5 | 32,753 (51.8%) | 27,050 (53.2%) | 5,703 (46.3%) | <0.001 |
| 6-10 | 13,696 (21.7%) | 10,842 (21.3%) | 2,854 (23.2%) | <0.001 |
| >10 | 4,525 (7.2%) | 3,279 (6.5%) | 1,246 (10.1%) | <0.001 |

**Supplemental Table 4**. Multivariable analysis of post-operative outcomes among surgical patients by number of PC appointments in the year prior to surgery, stratified by comorbidity status.

| **Outcome** | **No Comorbidities** | **p-value** | **Comorbidities** | **p-value** |
| --- | --- | --- | --- | --- |
| Textbook outcome |  |  |  |  |
| None | Ref | - | Ref | - |
| 1-5 | 1.21 [1.15–1.27] | **<.0001** | 1.18 [1.06–1.31] | **0.0017** |
| 6-10 | 1.06 [1.00–1.24] | 0.0653 | 1.08 [0.96–1.22] | 0.1869 |
| >10 | 0.96 [0.88–1.04] | 0.3063 | 0.94 [0.81–1.09] | 0.3853 |
| Complication |  |  |  |  |
| None | Ref | - | Ref | - |
| 1-5 | 0.85 [0.80–0.91] | **<.0001** | 0.91 [0.81–1.02] | 0.1020 |
| 6-10 | 0.94 [0.87–1.02] | 0.1111 | 0.96 [0.85–1.09] | 0.5489 |
| >10 | 0.96 [0.86–1.07] | 0.4685 | 0.99 [0.84–1.16] | 0.8881 |
| Prolonged length of stay |  |  |  |  |
| None | Ref | - | Ref | - |
| 1-5 | 0.79 [0.74–0.84] | **<.0001** | 0.84 [0.75–0.94] | **0.0024** |
| 6-10 | 0.84 [0.78–0.90] | **<.0001** | 0.81 [0.71–0.92] | **0.0015** |
| >10 | 0.87 [0.78–0.97] | **0.0098** | 0.94 [0.79–1.11] | 0.4346 |
| 90-day readmission |  |  |  |  |
| None | Ref | - | Ref | - |
| 1-5 | 0.87 [0.83–0.92] | **<.0001** | 0.92 [0.83–1.02] | 0.1047 |
| 6-10 | 1.01 [0.95–1.08] | 0.7299 | 1.02 [0.90–1.14] | 0.7949 |
| >10 | 1.16 [1.06–1.28] | **0.0012** | 1.22 [1.06–1.42] | **0.0067** |
| 90-day mortality |  |  |  |  |
| None | Ref | - | Ref | - |
| 1-5 | 0.81 [0.72–0.92] | **0.0009** | 0.91 [0.76–1.10] | 0.3263 |
| 6-10 | 0.89 [0.77–1.02] | 0.1020 | 0.87 [0.70–1.08] | 0.1953 |
| >10 | 0.92 [0.75–1.12] | 0.4016 | 0.92 [0.70–1.19] | 0.5120 |
